# Phytochemical Characterization, Antioxidant Activity, and Anti-Melanoma Mechanism of Flower Buds of *Magnolia biondii* Pamp.

**DOI:** 10.3390/plants14111725

**Published:** 2025-06-05

**Authors:** Shanshan Li, Gege Jiao, Penghui Ou, Xiaona Zhang, Yang Yu, Yihui Wang, Qingping Yao, Wei Wang

**Affiliations:** 1 School of Perfume & Aroma and Cosmetics, Shanghai Institute of Technology, Shanghai 201418, China; liss@mail.sit.edu.cn (S.L.); 226071132@mail.sit.edu.cn (G.J.); 226072143@mail.sit.edu.cn (P.O.); 226071147@mail.sit.edu.cn (X.Z.); 226072151@mail.sit.edu.cn (Y.Y.); 226072147@mail.sit.edu.cn (Y.W.); 2 Institute of Mechanobiology & Medical Engineering, School of Life Sciences & Biotechnology, Shanghai Jiao Tong University, Shanghai 200240, China; qpyao@sjtu.edu.cn

**Keywords:** the flower buds of *Magnolia biondii* Pamp., *Flos Magnoliae*, melanoma, network pharmacology, phytochemistry, mechanism, JAK/STAT signaling pathway

## Abstract

The flower buds of *Magnolia biondii* Pamp. (MBP), one of the botanical sources of *Xinyi* (*Flos Magnoliae*), are widely used in traditional medicine; however, their potential role in melanoma treatment remains unexplored. In this study, the phytochemical composition, antioxidant activity, and anti-melanoma mechanisms of MBP extracts were systematically investigated. Phytochemical profiling using UHPLC-Q-Exactive Orbitrap MS identified 26 bioactive compounds. The ethanol extract exhibited high total flavonoid and polyphenol contents, correlating with enhanced antioxidant capacity as demonstrated by DPPH and ABTS assays. Network pharmacology analysis highlighted the JAK/STAT signaling pathway, identifying STAT3 and STAT1 as core targets. Western blot analysis confirmed MBP significantly inhibited the phosphorylation of JAK1 and STAT1 in melanoma cells. Connectivity Map (CMap) and network analyses further pinpointed naringenin as a primary active constituent. In vitro assays demonstrated that MBP and naringenin inhibited the proliferation and migration of A375 and B16F10 melanoma cells, while exhibiting relatively low cytotoxicity toward normal keratinocytes. Molecular docking and dynamics simulations revealed strong and stable binding interactions between naringenin and JAK1/STAT1 proteins. These findings collectively support MBP and naringenin as promising candidates for melanoma treatment, providing mechanistic evidence for their targeted activity and laying a foundation for future research and clinical applications.

## 1. Introduction

Malignant melanoma is a highly aggressive form of skin cancer that can arise in any tissue containing melanocytes [1]. Despite representing only 0.05% of global annual skin cancer cases, melanoma is the most lethal type, responsible for the majority of skin cancer-related deaths (World Health Organization), making it a considerable public health challenge worldwide. Traditional treatments are often hindered by issues such as drug resistance and adverse side effects [2], underscoring the urgent need for novel therapeutic strategies. Natural compounds have gained attention as promising cancer treatments due to their safety and low toxicity profiles [3]. For instance, curcumin and paclitaxel have been extensively studied and are proven to possess potent anticancer properties [4,5].

**Figure 1:**
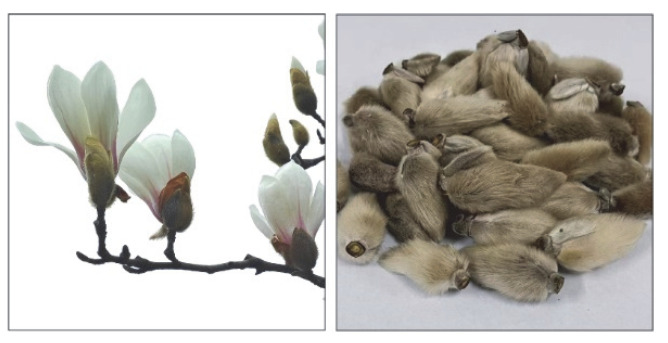
The flowers (**left**) and flower buds (**right**) of MBP.

Plant-derived compounds, while primarily serving as adaptive mechanisms for environmental response [6], have also been shown to activate biochemical pathways in humans and animals. This highlights their potential for preventing and treating melanoma, making plant extracts valuable resources for developing innovative therapies. Among these, the dried flower buds of *Magnolia biondii* Pamp. (MBP), also known as *Flos Magnoliae* or *Xinyi* [7], are shown in Figure 1. In traditional Chinese medicine, *Xinyi* refers to the flower buds of several Magnolia species, including *Magnolia biondii* Pamp., *Magnolia denudate* Desr., and *Magnolia sprengeri* Pamp [8]. Among these, *MBP* is the most widely used species in clinical and market settings, and was selected as the plant material in this study [9]. First documented in Li Shizhen’s renowned Ming Dynasty text *Ben Cao Gang Mu* (1578 A.D.), *Xinyi* has been widely used in traditional medicine to treat conditions such as abscesses, allergic rhinitis, and nasal congestion [10]. *Xinyi* comprises diverse bioactive compounds, including lignans, essential oils, and polysaccharides [11,12,13]. Recent studies indicate *Xinyi* has antioxidant, anti-inflammatory, anti-allergic, anti-diabetic, and ovarian function-preserving properties [14,15,16,17,18]. Despite *Xinyi*’s established therapeutic uses, MBP’s potential in melanoma treatment remains largely unexplored, warranting further investigation to uncover its active components and mechanisms of action.

Melanoma, like many human diseases, involves complex interactions among multiple genes, proteins, and signaling pathways, making it particularly challenging to treat. Therefore, traditional Chinese medicine (TCM), with its “multi-component” and “multi-target” mechanisms, offers a promising approach for addressing these complexities [19]. Network pharmacology, a novel methodology integrating biological network analysis and target prediction, provides a systematic framework to study these mechanisms [20]. Furthermore, UHPLC-Q-Exactive Orbitrap MS, a high-resolution analytical technique, complements network pharmacology by enabling the precise identification and characterization of complex phytochemicals present in plants [21]. Through accurate mass measurements and structural analysis, it facilitates the discovery of bioactive compounds that may act on multiple targets and pathways. This combined approach, utilizing comprehensive compound profiling along with network analysis, allows for a deeper understanding of the mechanisms underlying TCM-based therapies. By integrating these techniques, valuable insights into key drug targets and pathways can be systematically identified, providing a robust foundation for elucidating the therapeutic mechanisms of botanical TCM in disease treatment [22,23,24].

In this study, a UHPLC-Q-Exactive Orbitrap MS-based widely targeted metabolomics approach was employed to comprehensively profile the chemical constituents of MBP. By integrating metabolomics with network pharmacology, we elucidated the potential mechanisms through which MBP exerts its anti-melanoma effects. Bioinformatics and topological analyses further identified the key targets and pathways modulated by MBP, findings that were subsequently validated via in vitro experiments demonstrating that both MBP and its active compound, naringenin, effectively inhibit melanoma cell proliferation and migration. These findings deepen our understanding of MBP’s active constituents and therapeutic potential while providing a foundation for its development as a natural anticancer agent.

## 2. Results

### 2.1. Quantitative Analysis of Total Flavonoid and Polyphenol Contents

The total flavonoid content (TFC) and total polyphenol content (TPC) of *Magnolia biondii* Pamp. (MBP) flower bud extracts obtained using water and 80% ethanol are summarized in Table 1. The ethanol extract exhibited significantly higher TFC (123.81 mg RE/g extract) compared to the water extract (67.55 mg RE/g extract), indicating that ethanol was more effective in extracting flavonoids. A similar trend was observed in TPC values, where the ethanol extract yielded 10.79 mg GAE/g extract, higher than the 6.53 mg GAE/g extract obtained from the water extract. These results suggest that 80% ethanol is a more efficient solvent for extracting both flavonoids and polyphenols from MBP.

### 2.2. Qualitative Detection of Phytochemical Constituents

Following the quantification of the total flavonoid and phenolic contents, a comprehensive chemical analysis of MBP was carried out using UHPLC-Q-Exactive Orbitrap MS. A total of 26 compounds were identified based on their retention times, accurate *m*/*z* values, adduct forms, and MS/MS fragmentation patterns, as summarized in Table 2. The TIC chromatograms in both positive and negative ionization modes are shown in Figure S1, and the corresponding MS/MS spectra of representative compounds are provided in Figure S2. To enhance structural annotation, MS/MS spectral matching was also performed using the Global Natural Products Social Molecular Networking (GNPS) platform. Matches with cosine similarity scores greater than 0.7 were selected as high-confidence identifications [25,26], and mirror plots of representative matches are presented in Figure S3. Compound identities were further supported by comparison with reported data in the literature under similar chromatographic conditions and by matching to entries in established spectral databases. These results collectively establish a detailed phytochemical profile of MBP, providing a chemical basis for subsequent biological activity analysis.

### 2.3. In Vitro Antioxidant Activity of MBP

The antioxidant activity of *Magnolia biondii* Pamp. (MBP) flower bud extracts was evaluated using DPPH and ABTS radical scavenging assays (Table 3). The ethanol extract exhibited significantly lower IC_50_ values—88.14 μg/mL (DPPH) and 100.22 μg/mL (ABTS)—compared to 115.77 μg/mL and 458.27 μg/mL for the water extract, respectively. Overall, both extracts demonstrated moderate free radical scavenging ability, with the ethanol extract showing notably stronger antioxidant potential.

### 2.4. Anti-Proliferative Effect of MBP on Melanoma Cells

**Figure 2:**
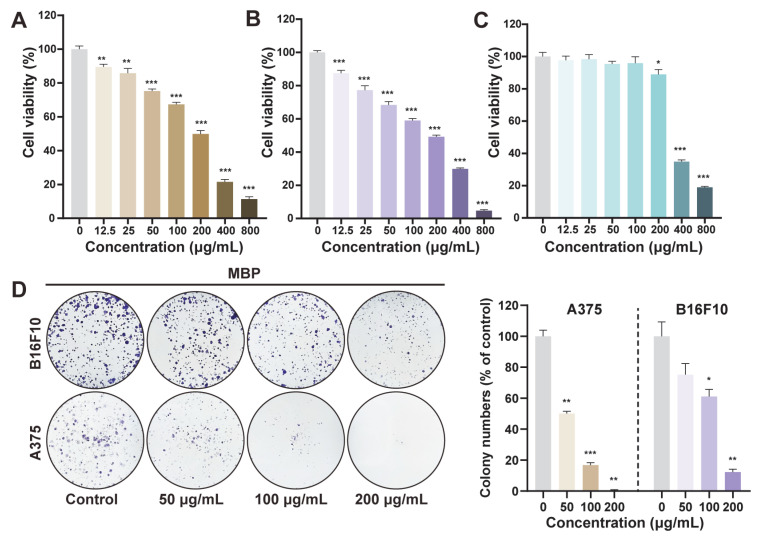
Inhibition of cell viability and clonogenicity by MBP. (**A**–**C**) Cell viability of A375 (**A**), B16F10 (**B**), and HaCaT (**C**) cells treated with MBP (0–800 μg/mL) assessed by CCK-8. (**D**) Colony formation of A375 and B16F10 cells following MBP treatment (0, 50, 100, and 200 μg/mL). Representative images (left) and quantification (right) are shown. Data are presented as mean ± SEM (*n* = 3). * *p* < 0.05, ** *p* < 0.01, *** *p* < 0.001 vs. control.

The anti-proliferative effects of MBP were evaluated using CCK-8 and colony formation assays. The CCK-8 results revealed that MBP extract inhibited the proliferation of both B16F10 mouse melanoma cells and A375 human melanoma cells in a concentration-dependent manner across the range of 12.5–800 μg/mL (Figure 2A,B). In contrast, normal human HaCaT keratinocytes maintained over 90% viability within the 12.5–200 μg/mL range (Figure 2C), indicating that MBP preferentially affects melanoma cells while having lower cytotoxic effects on non-cancerous cells. Based on these findings, concentrations up to 200 μg/mL were chosen for subsequent experiments, supporting MBP’s potential as a candidate therapeutic agent with a reduced risk of side effects [29]. Colony formation assays further confirmed MBP’s inhibitory effects, showing a significant reduction in the clonogenic potential of both melanoma cell lines (Figure 2D), suggesting the suppression of long-term proliferative potential.

### 2.5. Anti-Migratory Effect of MBP on Melanoma Cells

**Figure 3:**
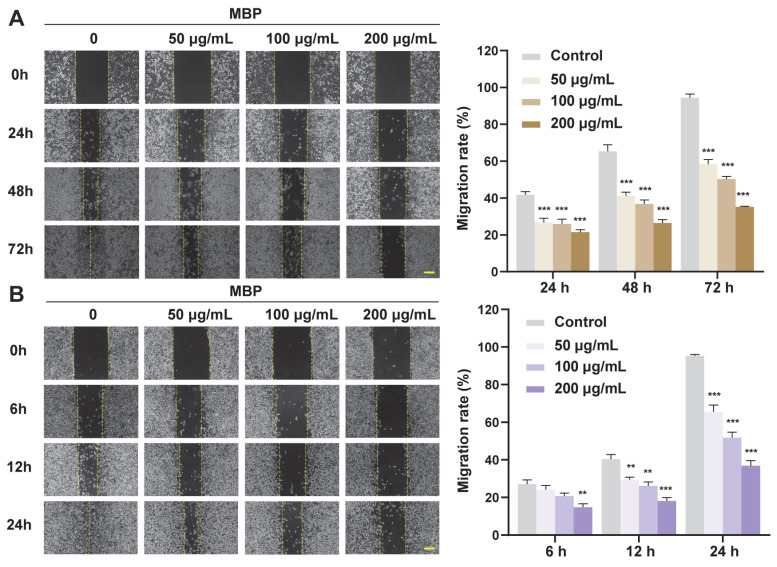
Inhibition of cell migration by MBP. (**A**,**B**) Wound healing assays of A375 (**A**) and B16F10 (**B**) cells treated with MBP (0, 50, 100, and 200 μg/mL). Migration was monitored over time, and representative images (left) and quantification (right) are shown. Scale bar: 200 μm. Data are presented as mean ± SEM (*n* = 3). ** *p* < 0.01, *** *p* < 0.001 vs. control.

The anti-migratory effect of MBP was assessed via wound healing assays (Figure 3). Treatment with MBP significantly reduced the migration of both B16F10 and A375 melanoma cell lines. Over time, MBP-treated cells exhibited markedly slower wound closure compared to untreated controls, indicating impaired migratory capacity [30]. These findings suggest that MBP may potentially interfere with melanoma cell metastasis by limiting their ability to migrate.

### 2.6. Network Analysis of MBP Targets in Melanoma Treatment

#### 2.6.1. Predicted Targets of MBP

**Figure 4:**
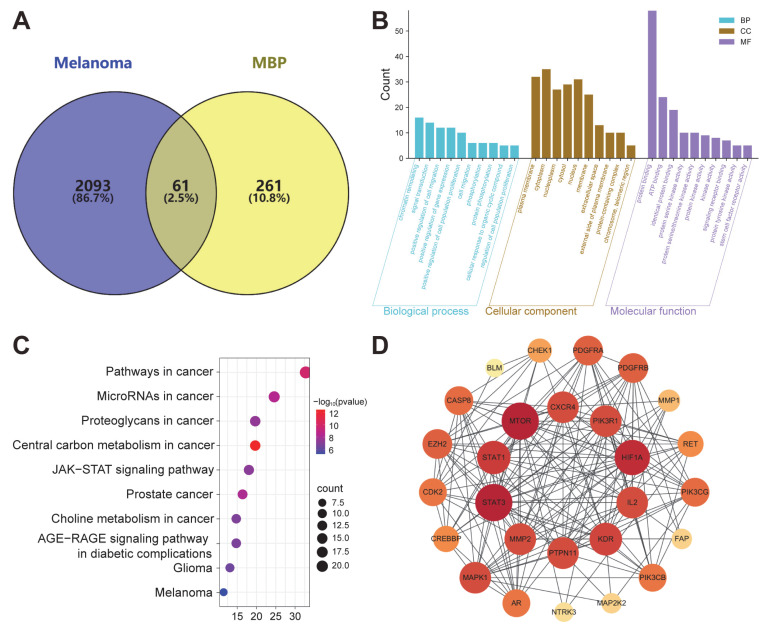
Network pharmacology analysis of MBP in melanoma. (**A**) A Venn diagram showing the overlapping targets between melanoma-related genes and MBP-predicted targets. (**B**) GO enrichment analysis of common targets, including biological processes (BPs), cellular components (CCs), and molecular functions (MFs). The top 10 terms in each category are shown. (**C**) KEGG pathway enrichment analysis of overlapping targets. The top 10 significantly enriched pathways are presented. (**D**) A PPI network constructed from overlapping targets. The node color intensity reflects the degree value: red indicates a higher degree, and lighter colors represent lower degrees.

Based on the UHPLC-MS/MS analysis, 322 potential targets of MBP were predicted from 26 active compounds using the SuperPred database. To identify melanoma-related targets, 2154 genes were retrieved from the GeneCards, OMIM, and GEO databases. After removing duplicates, these melanoma-related genes were intersected with the 322 MBP targets, yielding 61 overlapping genes, which were recognized as potential therapeutic targets for MBP in melanoma treatment (Figure 4A).

#### 2.6.2. GO and KEGG Analysis

To investigate the potential mechanisms of MBP in melanoma treatment, we conducted GO and KEGG enrichment analyses to identify melanoma-related biological processes (BPs), cellular components (CCs), and molecular functions (MFs) associated with the identified targets. The top 10 enriched terms in each category are shown in Figure 4B. Prominent BP terms such as “cell proliferation”, “cell migration”, and “protein phosphorylation” were significantly enriched, which are closely associated with melanoma progression. CC analysis identified essential intracellular components, including the cytoplasm, nucleus, and plasma membrane, as primary sites of action for MBP. In terms of MF, the enriched terms included “protein binding”, “protein kinase activity”, and “signaling receptor binding”.

Notably, KEGG analysis highlighted the JAK-STAT signaling pathway as a key mechanism, along with several other cancer-related pathways (Figure 4C). Given these findings, this study strongly suggests that MBP may exert its therapeutic effects on melanoma through the modulation of the JAK-STAT pathway, which was prioritized for further experimental validation.

#### 2.6.3. PPI Analysis

The melanoma-related targets of MBP were integrated into the STRING database to analyze protein–protein interactions. The data collected from the STRING database was rebuilt using the Cytoscape program, resulting in a PPI network with 51 nodes and 283 edges (Figure 4D). The top ten core targets were filtered based on their degree value (Table 4). Notably, STAT3 and STAT1 were among the top-ranked targets, further supporting the potential involvement of the JAK-STAT signaling pathway in MBP’s anti-melanoma activity. Future investigation is needed into whether MBP can directly modulate JAK and STAT activation and lead to enhanced tumor suppression in melanoma.

#### 2.6.4. “Component-Disease-Target” Network Construction

**Figure 5:**
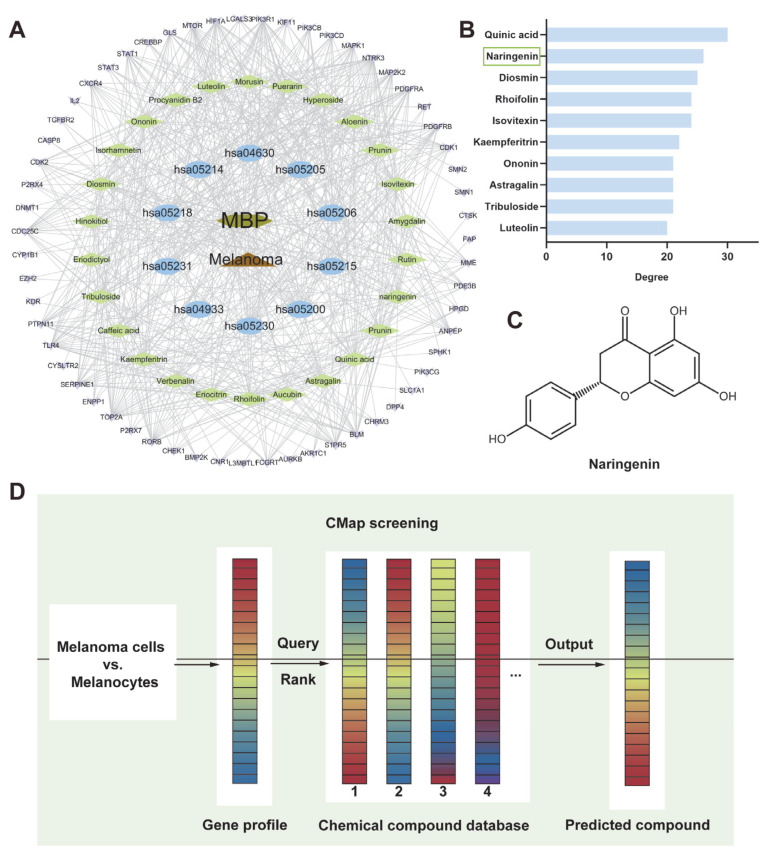
The prediction of key active compounds in MBP against melanoma. (**A**) A “Component–Disease–Target” network illustrating the interactions between MBP-derived compounds, melanoma-related targets, and associated pathways. (**B**) Top-ranked MBP compounds identified based on degree centrality in the network. (**C**) The chemical structure of naringenin. (**D**) A schematic diagram of the Connectivity Map (CMap) analysis workflow for compound screening.

To identify key anti-melanoma compounds in MBP, a “Component–Disease–Target (C-D-T)” network was constructed based on 61 overlapping genes, yielding 97 nodes and 627 edges (Figure 5A). Quinic acid, naringenin, and diosmin were ranked as the top three candidates based on degree centrality (Figure 5B), suggesting that they may be key contributors to the observed therapeutic effects.

#### 2.6.5. Identification of Primary Anti-Melanoma Active Compounds Using CMap

Connectivity Map (CMap) analysis was conducted to prioritize active compounds with potential therapeutic relevance. The top 10 compounds with connectivity scores ≤ −0.4 are summarized in Table 5. Naringenin, glycitein, and eugenol were identified as the top three phytochemical compounds with negative connectivity scores. Among them, naringenin showed the most pronounced inverse correlation, with a connectivity score of −0.47, suggesting potential anti-melanoma efficacy. Together with its high network centrality, naringenin was selected for further validation (Figure 5C). The screening workflow is shown in Figure 5D.

### 2.7. Validation of JAK1/STAT1 Pathway Involvement in MBP-Treated Melanoma Cells

**Figure 6:**
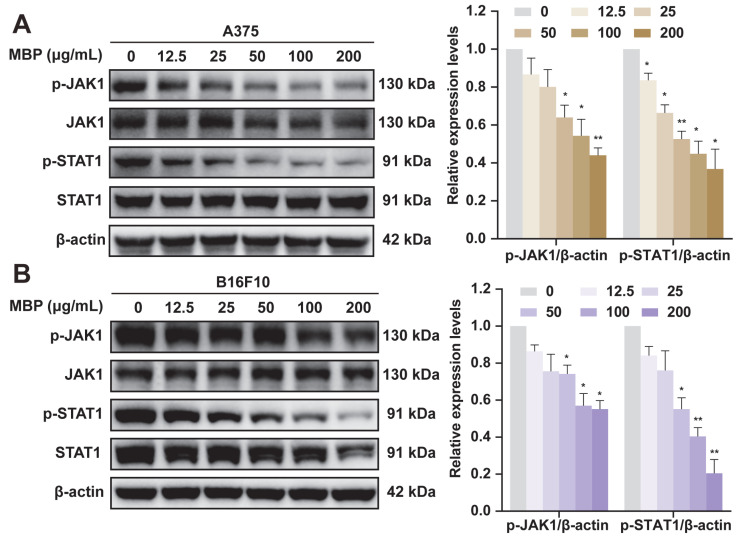
The effects of MBP on JAK1/STAT1 signaling in melanoma cells. (**A**,**B**) A375 (**A**) and B16F10 (**B**) cells were treated with MBP (0–200 μg/mL) for 24 h. The protein levels of JAK1, p-JAK1, STAT1, and p-STAT1 were analyzed by Western blot analysis. β-Actin served as the loading control. The relative expression of phosphorylated proteins is quantified. Data are presented as the mean ± SEM (*n* = 3). * *p* < 0.05, ** *p* < 0.01 vs. control.

Through network pharmacological analysis, the JAK/STAT signaling pathway was identified as a critical mechanism for melanoma treatment, with STAT1 highlighted as a key target. Based on these findings, the role of the JAK1/STAT1 pathway in MBP-treated melanoma cells was experimentally validated by Western blot analysis. In untreated cells, high levels of phosphorylated JAK1 (p-JAK1) and STAT1 (p-STAT1) were observed. MBP treatment led to a dose-dependent reduction in both p-JAK1 and p-STAT1 levels, while the total JAK1 and STAT1 protein levels remained largely unchanged (Figure 6A,B). These findings are consistent with the results from the network pharmacology analysis, which predicted the JAK1/STAT1 signaling pathway as a critical mechanism for MBP’s therapeutic effects.

### 2.8. Anti-Melanoma Activity of Naringenin

**Figure 7:**
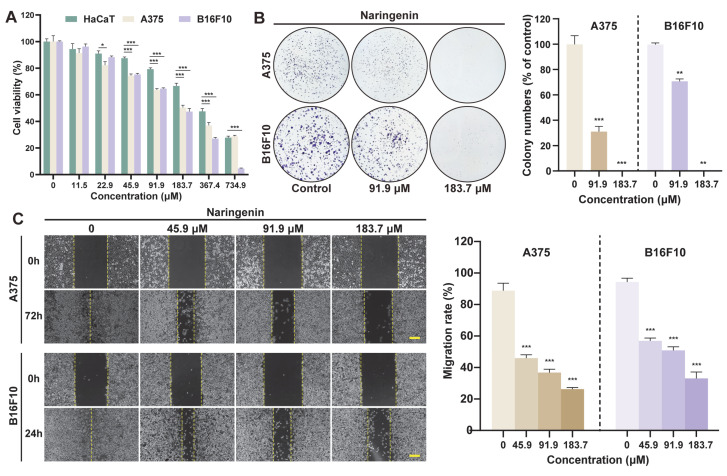
Effects of naringenin on melanoma cell viability, clonogenicity, and migration. (**A**) CCK-8 assay of HaCaT, A375, and B16F10 cells treated with naringenin. (**B**) Colony formation of A375 and B16F10 cells after treatment with naringenin. Representative images (left) and quantification (right) are shown. (**C**) Wound healing assay of A375 (0–72 h) and B16F10 (0–24 h) cells treated with naringenin. Scale bar: 200 μm. Data are presented as mean ± SEM (*n* = 3). * *p* < 0.05, ** *p* < 0.01, *** *p* < 0.001 vs. control.

To further investigate the bioactive compounds of MBP, naringenin was evaluated for its effects on B16F10 and A375 melanoma cells over a 24 h period at various concentrations. Figure 7A illustrates that naringenin inhibited the growth of both B16F10 and A375 cells in a dose-dependent manner, with approximately 50% inhibition observed at 183.7 μM. At this concentration, naringenin exhibited comparatively lower cytotoxicity in normal HaCaT keratinocytes, suggesting a degree of differential sensitivity between cancerous and non-cancerous cells. A subsequent colony formation assay confirmed these findings, with a significant reduction in colony numbers observed in both A375 and B16F10 cells at higher doses (Figure 7B). Additionally, the wound healing assay indicated a dose-dependent decrease in cell migration, further supporting naringenin’s role in impeding tumor cell motility (Figure 7C). Taken together, these findings suggest that naringenin may contribute to the overall anti-melanoma activity observed in MBP, potentially through multiple mechanisms.

### 2.9. Molecular Docking Analysis

Building on the hypothesis, molecular docking was further conducted to evaluate the binding interactions of naringenin with two key targets in the JAK-STAT pathway, JAK1 and STAT1. The FDA-approved JAK inhibitors ruxolitinib and tofacitinib were used as reference compounds, as both known to suppress JAK1 activity and downstream STAT1 signaling [31]. Docking simulations were carried out using AutoDock Vina, and binding energies were assessed to reflect ligand–protein interaction strength. Generally, docking scores below −5.0 kcal/mol indicate moderate affinity, while values below −7.0 kcal/mol suggest strong and stable binding [32].

**Figure 8:**
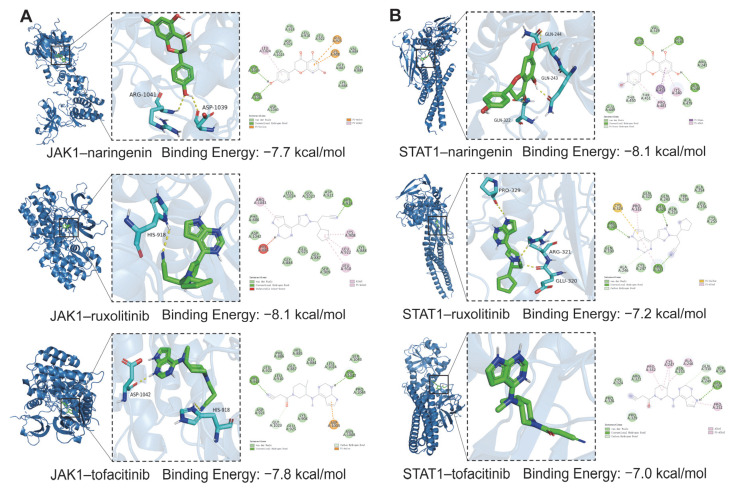
Molecular docking interactions of JAK1 and STAT1 with naringenin and reference inhibitors. (**A**) The 3D binding poses and 2D interaction diagrams of JAK1 with naringenin, ruxolitinib, and tofacitinib. (**B**) The 3D binding poses and 2D interaction diagrams of STAT1 with naringenin, ruxolitinib, and tofacitinib.

As shown in Figure 8A, naringenin exhibited a binding energy of −7.7 kcal/mol with JAK1, stabilized by hydrogen bonding with ASP1039 and ARG1041. This interaction was comparable to that of ruxolitinib (−8.1 kcal/mol) and tofacitinib (−7.8 kcal/mol). With STAT1 (Figure 8B), naringenin demonstrated an even lower binding energy of −8.1 kcal/mol, forming hydrogen bonds with GLN243, GLN244, and GLN322, indicating higher predicted affinity than the reference compounds (ruxolitinib: −7.2 kcal/mol; tofacitinib: −7.0 kcal/mol).

These findings highlight the potential of naringenin to bind stably with both JAK1 and STAT1, supporting its proposed involvement in modulating the JAK-STAT signaling axis and contributing to the observed anti-melanoma activity of MBP.

### 2.10. Molecular Dynamics Simulation Analysis

To further investigate the structural stability and dynamic behavior of naringenin bound to JAK1 and STAT1, 100 ns molecular dynamics (MDs) simulations were performed. Multiple parameters, including root-mean-square deviation (RMSD), the radius of gyration (Rg), the solvent-accessible surface area (SASA), hydrogen bonds (H-bonds), root-mean-square fluctuation (RMSF), and binding free energy, were assessed to evaluate the conformational stability and binding affinity of the protein–ligand complexes.

**Figure 9:**
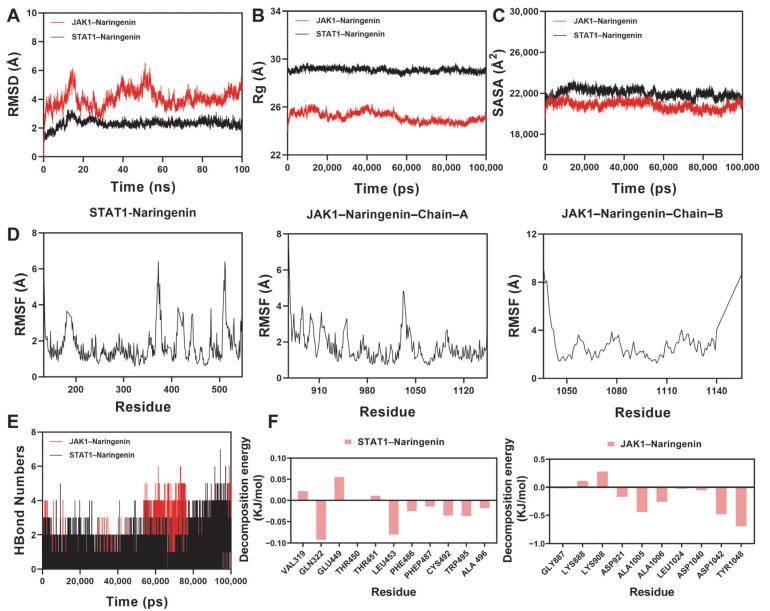
Molecular dynamics simulation of JAK1–naringenin and STAT1–naringenin complexes. (**A**) RMSD profiles; (**B**) radius of gyration (Rg); (**C**) solvent-accessible surface area (SASA); (**D**) root-mean-square fluctuation (RMSF) per residue; (**E**) number of hydrogen bonds over time; and (**F**) MM/PBSA per-residue binding energy decomposition.

As illustrated in Figure 9A, the RMSD values indicated that the JAK1–naringenin complex reached equilibrium after approximately 75 ns, stabilizing at ~4.7 Å, while the STAT1–naringenin complex stabilized earlier, at around 20 ns, with fluctuations centered near 2.4 Å. These trends suggest high structural stability, particularly in the STAT1 complex. Rg analysis (Figure 9B) reflected consistent structural compactness for both complexes. The Rg of JAK1–naringenin remained stable throughout the simulation, whereas STAT1–naringenin showed minor fluctuations before gradually stabilizing, suggesting slight conformational adjustments upon ligand binding. The SASA values of both complexes exhibited mild oscillations (Figure 9C), indicating subtle changes in solvent exposure that may result from binding-induced local structural rearrangements.

The RMSF values for most residues in both complexes were below 4 Å (Figure 9D), indicating limited local flexibility and further supporting the structural stability of the ligand-bound proteins. H-bond analysis (Figure 9E) revealed that the number of hydrogen bonds formed between naringenin and JAK1 ranged from 0 to 6, with an average of ~4. For the STAT1 complex, hydrogen bond numbers ranged from 0 to 7, also averaging around 4. This consistent interaction pattern suggests favorable hydrogen bonding, contributing to complex stability.

Subsequently, binding free energies were calculated using the Molecular Mechanics/Poisson–Boltzmann Surface Area (MM/PBSA) approach based on representative equilibrium conformations. The free energy of binding was estimated to be −54.458 kJ/mol for the JAK1–naringenin complex and −21.707 kJ/mol for the STAT1–naringenin complex, suggesting a stronger binding affinity to JAK1. Further per-residue energy decomposition identified key amino acid residues contributing to ligand binding (Figure 9F). In the JAK1 complex, TYR1048, ASP1042, ALA1005, and ALA1006 showed substantial energy contributions. In the STAT1 complex, GLN322, LEU453, CYS492, TRP495, and PHE486 were the main contributors. These residues are likely involved in stabilizing the ligand within the binding site and may be functionally relevant to the protein’s activity.

Taken together, these MD results demonstrate that naringenin forms energetically stable and structurally consistent complexes with both JAK1 and STAT1, with stronger affinity observed toward JAK1. This supports its potential role in modulating the JAK1–STAT1 signaling pathway.

## 3. Discussion

In the current study, the phytochemical analysis demonstrated that *Magnolia biondii* Pamp. (MBP) flower bud extracts contained significant amounts of total flavonoids and polyphenols, particularly when extracted with 80% ethanol. This solvent proved more efficient than water in extracting these bioactive components. The higher flavonoid and polyphenol contents observed in the ethanol extract likely contributed to its enhanced antioxidant capacity, as demonstrated by its lower IC_50_ values in the DPPH and ABTS assays. Flavonoids are well-established natural antioxidants that neutralize free radicals and oxidative stress, mechanisms closely linked to melanoma pathogenesis and progression [33]. Previous research has suggested that flavonoids exhibit anticancer effects by modulating oxidative stress-related pathways and inflammation, further supporting their potential therapeutic utility in melanoma [34,35].

UHPLC-Q-Exactive Orbitrap MS analysis identified 26 compounds within MBP, suggesting that its observed anti-melanoma activity may stem from synergistic interactions among multiple phytochemical constituents. Among these compounds, several flavonoids, including astragalin, rutin, naringenin, tribuloside, and kaempferitrin, have previously been reported for their anticancer effects. For example, astragalin demonstrates cytotoxic properties and apoptosis induction by modulating critical melanoma-associated proteins such as caspase-9/3 and SOX10 [36]. Similarly, rutin induces cell senescence and morphological alterations in SK-MEL-28 melanoma cells at elevated concentrations [37].

The experimental results demonstrated MBP’s anti-proliferative and anti-migratory effects on melanoma cells, accompanied by relatively low cytotoxicity to non-cancerous HaCaT keratinocytes. This evaluation accounted for differences in baseline proliferation rates by normalizing viability within each cell line, indicating its potential suitability for further investigation in skin-related applications. Both short-term proliferation assays and long-term colony formation experiments consistently revealed MBP’s capacity to suppress melanoma cell growth, supporting its potential as a candidate for further preclinical evaluation [38]. Additionally, wound healing assays indicated that MBP significantly inhibited melanoma cell migration, suggesting that MBP may potentially impede tumor metastasis.

To systematically elucidate the potential anti-melanoma mechanisms of MBP, a network pharmacology approach identified 61 overlapping genes as potential therapeutic targets. GO enrichment analysis revealed involvement in critical biological processes, including cell proliferation, migration, and protein phosphorylation, all of which are closely related to melanoma development. KEGG pathway enrichment analysis underscored the importance of the JAK-STAT signaling pathway, a well-characterized mediator of melanoma progression and immune evasion [39]. Moreover, the literature evidence supports the JAK/STAT pathway modulation of melanoma proliferation via multiple intracellular mechanisms; for example, Orlova et al. indicated interactions with the STAT5B-SH2 domain as a potential regulatory mechanism [40], while NF-κB/STAT3 pathway modulation has been associated with melanoma metastasis [41]. The potential involvement of the JAK2-NLRP3 axis, implicated in melanoma regulation and immune modulation, further emphasizes the complexity and breadth of MBP’s therapeutic actions by modulating JAK/STAT pathways [42]. Notably, while several studies have explored related signaling pathways, the specific impact of MBP on the JAK1/STAT1 axis remains underexplored, representing a promising direction for future research.

Consistent with these computational predictions, experimental validation via Western blot analysis demonstrated that MBP effectively downregulated phosphorylated levels of JAK1 and STAT1 proteins, further confirming the functional significance of this pathway. The identified targets were further validated through protein–protein interaction (PPI) network analysis, revealing STAT3 and STAT1 as core regulatory hubs. STAT3 plays a key role in tumorigenesis and metastasis by promoting inflammation and immune evasion [43,44]. The constitutive overexpression of STAT1 in melanoma cells promotes a pro-metastatic and therapy-resistant phenotype [45]. These observations reinforce JAK-STAT signaling as a crucial mediator of MBP’s therapeutic effects and provide a strong rationale for targeting these transcription factors in melanoma management.

Subsequent C-T-D network analyses highlighted quinic acid, naringenin, and diosmin as key bioactive candidates. Previous studies have shown that extracts rich in quinic acid exhibit significant anti-melanoma effects, including the inhibition of melanoma cell growth and the induction of apoptosis, supporting quinic acid’s role as a potential bioactive component in MBP’s therapeutic effects [46]. Similarly, naringenin inhibits the proliferation and migration of B16F10 and SK-MEL-28 melanoma cells by reducing the phosphorylation of ERK1/2, JNK, and MAPK, thereby exerting anti-melanoma effects [47]. Diosmin has also been shown to significantly reduce metastatic nodules and lower the growth and invasion indices in the B16F10 lung metastasis model, indicating its potent antimetastatic activity [48]. These previous reports further corroborate our findings, suggesting that these compounds may represent the primary active constituents within MBP.

Naringenin is a flavonoid that is widely reported to exert anti-inflammatory, antioxidant, and anticancer activities [49]. Focusing on naringenin, our data confirmed its anti-proliferative activity against melanoma cells relative to normal keratinocytes, aligning with its reported specificity and anticancer efficacy. Naringenin significantly suppressed melanoma cell colony formation and migration, underscoring its pivotal role in mediating MBP’s anti-melanoma activity. Molecular docking analyses further revealed the strong binding affinities of naringenin with key signaling proteins JAK1 and STAT1, comparable to established JAK1 inhibitors (ruxolitinib and tofacitinib), indicating its promising interaction profiles [50]. Subsequent molecular dynamics (MDs) simulations provided additional mechanistic evidence, demonstrating stable interactions, consistent hydrogen bonding, and energetically favorable binding with functionally important residues [51,52]. These combined computational and experimental findings highlight naringenin’s potential therapeutic relevance through the modulation of the JAK1–STAT1 signaling pathway, reinforcing its role as an active anti-melanoma constituent of MBP.

Overall, this study provides comprehensive evidence supporting MBP’s potential as an anti-melanoma therapeutic agent. The combined results from phytochemical analyses, antioxidant assays, in vitro experiments, network pharmacology predictions, and molecular docking strongly suggest that MBP, particularly its active constituent naringenin, effectively suppresses melanoma proliferation and migration through the modulation of the JAK-STAT signaling pathway. These findings establish a solid mechanistic foundation and highlight MBP’s promise for future therapeutic development and clinical translation.

Future studies should focus on the quantitative analysis and comprehensive structural validation of MBP constituents. In particular, the use of authentic standards, advanced fingerprinting techniques, and structure–activity relationship models will be essential to clarify which compounds, or combinations thereof, are primarily responsible for the observed biological effects. In parallel, in vivo pharmacodynamic studies and animal models should be incorporated to evaluate efficacy, toxicity, and systemic responses. Together, these efforts will provide a stronger basis for evaluating MBP’s clinical relevance.

## 4. Materials and Methods

### 4.1. Chemicals and Reagents

Methanol, acetonitrile, formic acid, and isopropyl alcohol, all of the LC-MS grade, were supplied by ANPEL (Shanghai, China). Ultrapure water was generated using a Milli-Q water purification system (Millipore, Bedford, MA, USA). Naringenin (purity > 98%) was sourced from NatureStandard (Shanghai, China). Cell Counting Kit-8 (CCK-8) reagents and the anti-β-actin antibody (1:10,000) were provided by ShareBio (Shanghai, China). Antibodies targeting JAK1 (1:1000), phosphorylated JAK1 (1:1000), STAT1 (1:1000), and phosphorylated STAT1 (1:1000) were purchased from Cell Signaling Technology (CST, Boston, MA, USA).

### 4.2. Plant Materials

The flower buds of *Magnolia biondii* Pamp. (MBP) were collected in Nanzhao County, Nanyang City, Henan Province, China (33.4885° N, 112.4345° E) during November to December. The collection was assisted by local field staff familiar with regional plant morphology to ensure species accuracy. The fresh flower bud samples were freeze-dried at −50 °C for 48 h, and then ground into fine powder for subsequent phytochemical and biological experiments.

The extraction conditions (solvent concentration, temperature, time, and solid–liquid ratio) were optimized based on preliminary experiments. For ethanol extraction, 5 g of the powdered sample was extracted with 250 mL of 80% ethanol (*v*/*v*) at 64 °C for 41 min using an ultrasonic bath (200 W, 40 kHz), with a solid-to-solvent ratio of 1:50 (*w*/*v*). For aqueous extraction, 5 g of the sample was extracted with 175 mL of distilled water at 60 °C for 40 min under the same ultrasonic conditions, with a ratio of 1:35 (*w*/*v*). The extracts were filtered through filter paper and concentrated under reduced pressure at 38 °C using a rotary evaporator. The concentrated crude extracts were then freeze-dried and stored at −20 °C until further use. The average extraction yields were 15% for the ethanol extract and 9% for the water extract.

### 4.3. Quantitative Phytochemical Analysis

The contents of total flavonoids (TFC) and total polyphenols (TPC) in MBP were analyzed using a previously reported method with minor adjustments [53,54]. The TFC was quantified in terms of rutin equivalents per gram of the dry plant material (mg RE/g d.w.), while the TPC was measured as gallic acid equivalents (mg GAE/g d.w.).

### 4.4. Qualitative Analysis Based on UHPLC-Q Exactive™ HFX-LC-MS/MS

Qualitative analysis was conducted using an ultra-high-performance liquid chromatography coupled with a hybrid quadrupole orbitrap mass spectrometer (UHPLC-Q Exactive HFX, Thermo Fisher Scientific, Waltham, MA, USA). The system was equipped with a Waters HSS T3 column (100 × 2.1 mm, 1.8 μm) maintained at a column temperature of 40 °C. The mobile phase consisted of Milli-Q water with 0.1% formic acid (phase A) and acetonitrile with 0.1% formic acid (phase B). The gradient elution program was as follows: 0 min, phase A/phase B (100:0, *v*/*v*); 1 min, phase A/phase B (100:0, *v*/*v*); 12 min, phase A/phase B (5:95, *v*/*v*); 13 min, phase A/phase B (5:95, *v*/*v*); 13.1 min, phase A/phase B (100:0, *v*/*v*); and 17 min, phase A/phase B (100:0, *v*/*v*). The injection volume was set at 2 μL with a flow rate of 0.3 mL/min.

High-resolution mass spectrometry (HRMS) data were obtained using a Q Exactive HFX hybrid Quadrupole Orbitrap mass spectrometer (Thermo Fisher Scientific) with a heated electrospray ionization (ESI) source which was operated in the Full-MS-ddMS^2^ acquisition mode. The ESI source parameters were configured as follows: sheath gas pressure, 40 arbitrary units; auxiliary gas pressure, 10 arbitrary units; spray voltage, +3000 V (positive mode)/−2800 V (negative mode); ion source temperature, 350 °C; and ion transfer tube temperature, 320 °C. The scanning range of the primary mass spectrometry was 70–1050 Da, with a primary resolution of 70,000 and a secondary resolution of 17,500.

### 4.5. Invitro Antioxidant Activity Evaluation

#### 4.5.1. DPPH Radical Scavenging Assay

The DPPH radical scavenging activity of the extracts was evaluated according to a previously reported method with slight modifications [55]. A 0.1 mM DPPH solution was prepared in methanol, and 1 mL of this solution was mixed with 1 mL of the sample extract at varying concentrations. The mixture was incubated in the dark at room temperature for 30 min. The absorbance was then measured at 517 nm using a UV-Vis spectrophotometer. The percentage of DPPH radical scavenging was calculated according to the following equation:

(1) $$\%Inhibition=(1-\frac{{A_{Sample-}A}_{Blank}}{A_{Control}})\times 100$$

#### 4.5.2. ABTS Radical Scavenging Assay

The ABTS radical scavenging activity was assessed based on the method of [56], with minor modifications. The ABTS radical cation (ABTS•^+^) was generated by mixing 7 mM of the ABTS solution with 2.45 mM potassium persulfate and allowing the mixture to react in the dark at room temperature for 12–16 h. The resulting solution was diluted with methanol to an absorbance of 0.70 ± 0.02 at 734 nm. Then, 250 μL of the sample extract was added to 1 mL of the diluted ABTS•^+^ solution. After 6 min of incubation at room temperature, the absorbance was recorded at 734 nm. The percentage of radical inhibition was calculated in Equation (1).

### 4.6. Cell Culture

A375, B16F10, and HaCaT cells were obtained from the Cell Bank of the Chinese Academy of Sciences (Shanghai, China) and cultured in Dulbecco’s Modified Eagle Medium (DMEM, Solarbio, Beijing, China) supplemented with 10% fetal bovine serum and 1% penicillin–streptomycin (ShareBio, Shanghai, China). The cells were incubated at 37 °C in a humidified atmosphere with 5% CO_2_. Upon reaching approximately 80% confluence, the cells were rinsed with phosphate-buffered saline (PBS) and harvested using 0.25% trypsin-EDTA for subsequent experiments.

### 4.7. Cell Viability Assay

In vitro cytotoxicity was assessed using the CCK-8 assay [57]. A375, B16F10, and HaCaT cells were seeded into 96-well plates at a density of 1 × 10^5^ cells per 100 µL per well. After overnight incubation, the cells were treated with varying concentrations of MBP (0–800 µg/mL) or naringenin (0–734.9 µM) at 37 °C for 24 h. DMEM-treated cells served as negative controls. At the end of the treatment period, 100 µL of fresh medium containing 10 µL of CCK-8 was added to each well, followed by incubation in the dark at 37 °C for 30 min. The absorbance was measured at 490 nm using a microplate reader.

The population doubling times for the cell lines used in this study were 20 h for A375, 17 h for B16F10, and 28 h for HaCaT, according to supplier datasheets and previous reports [58,59,60]. To minimize potential bias arising from these proliferation rate differences, all experiments were conducted at similar initial seeding densities, and drug treatment was initiated when cells reached approximately 80% confluence. Cell viability in each assay was normalized to the corresponding untreated control group of each cell line (set as 100%).

### 4.8. Colony Formation Assay

The colony formation assay was performed based on previously reported methods, with slight modifications [61]. A375 and B16F10 cells were seeded as single-cell suspensions into 6-well culture plates at a density of 500 cells per well. Following a 24 h incubation period, the cells were treated with MBP or naringenin at different concentrations for 48 h. The cultures were maintained for two weeks, with the medium refreshed every three days to support colony formation. After the culture period, colonies were fixed with 4% paraformaldehyde and stained with 0.1% crystal violet solution (Adamas Life, Shanghai, China) at room temperature for 10 min. The stained colonies were photographed and counted to assess colony formation.

### 4.9. Cell Migration Assay

The migratory ability of the cells was evaluated using a wound healing assay [62]. A375 and B16F10 cells were seeded into 12-well plates at a density of 3 × 10^5^ cells per well and incubated for 24 h. A vertical scratch was made in the center of each well using a 200 μL pipette tip. After washing with PBS, the cells were treated with MBP or naringenin for 24–72 h to assess wound closure. DMEM without extracts served as the control. Images of the migrating cells were captured using a digital camera attached to a ZEISS inverted microscope equipped with ZEN blue software (version 3.8; Carl Zeiss Microscopy GmbH, Jena, Germany). The wound area reduction in A375 and B16F10 cells was quantified using the ImageJ software (version 1.54f; National Institutes of Health, Bethesda, MD, USA). The rate of cell migration was calculated as the percentage of reduction in wound area compared to the initial wound size at 0 h.

### 4.10. Network Pharmacology Analysis

#### 4.10.1. Targets Prediction of MBP

The SMILES of the identified compounds were retrieved from the PubChem database (https://pubchem.ncbi.nlm.nih.gov, accessed on 15 October 2024) and input into SuperPred (https://prediction.charite.de/, accessed on 15 October 2024), a tool for structural similarity prediction, to identify potential gene targets [63]. Gene names and IDs were then validated using UniProt (https://www.uniprot.org/, accessed on 15 October 2024), with the species restricted to “Homo sapiens”.

#### 4.10.2. Screening of Potential Targets for Melanoma

Melanoma-related targets were identified by integrating data from GeneCards, OMIM, and GEO databases. Genes associated with melanoma were retrieved using the keyword “Melanoma” and were restricted to Homo sapiens. In GeneCards (https://www.genecards.org/, accessed on 25 October 2024) and OMIM (https://www.omim.org/, accessed on 25 October 2024), genes were ranked based on relevance scores, with top-ranked genes being selected for further analysis. Additionally, microarray gene expression profiles from the GEO database (GSE35388, https://www.ncbi.nlm.nih.gov/geo/, accessed on 9 October 2024) were analyzed using GEO2R (https://www.ncbi.nlm.nih.gov/geo/geo2r/, accessed on 9 October 2024) to identify differentially expressed genes (DEGs) between melanoma cells and normal melanocytes, applying thresholds of |log FC| > 2 and *p* < 0.05. After removing duplicates, the genes with a relevance score > 10 from GeneCards and OMIM were integrated with DEGs from GSE35388 to compile a comprehensive list of melanoma-related targets.

#### 4.10.3. GO Function and KEGG Pathway Enrichment Analysis of the Target

The Venny 2.1.0 tool (https://bioinfogp.cnb.csic.es/tools/venny/, accessed on 14 January 2025) was employed to overlap melanoma-associated targets with MBP-related targets. The common targets of MBP and melanoma were analyzed for gene ontology (GO) and Kyoto Encyclopedia of Enrichment of Genes and Genomes (KEGG) pathway enrichment using the DAVID database (https://david.ncifcrf.gov/, accessed on 14 January 2025). GO enrichment analysis was performed to classify the targets into biological processes (BPs), cellular components (CCs), and molecular functions (MFs), providing insights into their biological roles. Additionally, KEGG pathway enrichment analysis was conducted to predict the biological characteristics and clarify the primary signaling pathways associated with MBP–melanoma targets. Gene annotation and pathway catalogs were selected based on the criteria of *p* < 0.05 to ensure accurate enrichment analysis. Visualization of the GO and KEGG enrichment results was performed using the Bioinformatics online platform (https://www.bioinformatics.com.cn, accessed on 14 January 2025), an integrated data visualization and analysis portal.

#### 4.10.4. Construction and Analysis of Protein–Protein Interaction (PPI) Network

The targets of MBP for melanoma treatment were input into the STRING database (https://cn.string-db.org/, accessed on 19 January 2025), restricted to “Homo sapiens”, with a minimum interaction confidence score set at 0.4 [64]. Cytoscape 3.7.1 was used to visualize the PPI network diagram, with node size and color adjusted based on degree values [65]. Subsequently, the downloaded data were further analyzed in Cytoscape 3.7.1, and the top 10 core targets were identified using Network Analyzer based on degree values.

#### 4.10.5. Construction of “Component-Disease-Target” Network

To identify potential bioactive compounds in MBP for melanoma treatment, a “Component–Disease–Target” network was constructed using Cytoscape 3.7.1. This network integrated MBP-derived compounds with overlapping melanoma-related targets and relevant pathways, and key components were subsequently identified based on their degree values.

#### 4.10.6. CMap Screening

To identify key anti-melanoma compounds in MBP, the Connectivity Map (CMap) database (https://clue.io/, accessed on 9 October 2024) was queried using the top differentially expressed genes (|logFC| > 5) from melanoma cells versus normal melanocytes. Connectivity scores, ranging from −1 to 1, with positive values indicate greater similarity and negative values suggest that a compound can reverse disease-related transcriptional signatures, indicating therapeutic potential. Scores approaching −1 typically correlate with stronger inhibitory effects against the disease phenotype. Compounds with negative connectivity scores are considered to have potential therapeutic relevance by reversing disease-associated transcriptional signatures.

### 4.11. Western Blot Assay

B16F10 and A375 cells were treated with MBP for 24 h, and then lysed in a Radioimmunoprecipitation Assay (RIPA) buffer containing protease and phosphatase inhibitors for 15 min. After washing with PBS, the cells were harvested using cell scrapers. Following treatment with the loading buffer, the cell lysates were heated at 100 °C for 10 min. The proteins were separated using 8% sodium dodecyl sulfate polyacrylamide gel electrophoresis (SDS-PAGE); they were then transferred to PVDF membranes, and the protein expression levels were assessed using the ChemiDoc imaging system. The band density was quantified using the ImageJ software.

### 4.12. Molecular Docking

The structure of the naringenin was retrieved from the PubChem database (https://pubchem.ncbi.nlm.nih.gov/, accessed on 15 October 2024) and processed using AutoDockTools 1.5.7, with the resulting files saved in the PDBQT format. The 3D crystal structures of the target proteins, STAT1 (PDB ID: 7NUF) and JAK1 (PDB ID: 6GGH), were obtained from the Protein Data Bank (PDB, https://www.rcsb.org/, accessed on 15 October 2024). Water molecules and ligands were removed from the target proteins using PyMOL 2.6.0. Hydrogenation, charge distribution, and atomic type assignments were performed in AutoDockTools. Molecular docking was conducted using AutoDock Vina 1.1.2, with the default parameters, to predict the binding modes and affinities between naringenin and the target proteins. The docking results were ranked based on binding affinity (kcal/mol), and the best-scoring conformations were selected for subsequent analysis. Visualization of docking poses and protein–ligand interactions was performed using PyMOL 2.6.0 and Discovery Studio 2019, highlighting key hydrogen bonds and other non-covalent interactions between naringenin and critical amino acid residues.

### 4.13. Molecular Dynamics Simulation

Molecular dynamics simulations were performed using GROMACS 2022 for 100 ns to assess the stability of the JAK1–naringenin and STAT1–naringenin complexes. The CHARMM36 force field was applied to the proteins, and GAFF2 parameters were used for the ligands. The complexes were placed in a periodic cubic box solvated with TIP3P water molecules and were neutralized with counterions. Electrostatic interactions were handled using PME, with Verlet cutoff schemes for van der Waals and Coulomb interactions (cutoff = 1.0 nm). The systems underwent energy minimization, followed by 100 ps NVT and NPT equilibration (100,000 steps, coupling constant 0.1 ps), and were then simulated for 100 ns at 310 K and 1 bar. Trajectory analysis was conducted using GROMACS tools for RMSD, Rg, RMSF, SASA, and hydrogen bonding. MM/PBSA binding free energies were calculated using the g_mmpbsa package after system stabilization.

### 4.14. Statistics Analysis

GraphPad Prism (version 8.0, GraphPad Software, San Diego, CA, USA) was used for graphic visualization and statistical analysis. Statistical differences between the groups were analyzed using the one-way analysis of variance (ANOVA). The statistical significance was set at *p* < 0.05, and the results are presented as the mean ± SEM.

## 5. Conclusions

This study demonstrated that *Magnolia biondii* Pamp. (MBP) flower buds and their key active compound, naringenin, possess significant anti-melanoma activities. MBP, especially when extracted with 80% ethanol, exhibited high levels of flavonoids and polyphenols, correlating with its notable antioxidant capacity. Network pharmacology analysis combined with experimental validation indicated that MBP exerts its anti-melanoma effects through multiple targets and pathways, prominently involving the JAK1/STAT1 signaling pathway. Furthermore, naringenin was confirmed to be a primary bioactive component, selectively inhibiting melanoma cell proliferation, clonogenicity, and migration. These findings support the potential application of MBP and naringenin as functional foods or complementary therapeutic agents for melanoma. Future studies are required to further clarify their mechanisms and evaluate their clinical efficacy and safety.

## Figures and Tables

**Table 1:** Total flavonoid content (TFC) and total polyphenol content (TPC) of water and 80% ethanol MBP extracts. RE: rutin equivalents; GAE: gallic acid equivalents.

Solvent	TFC (mg RE/g Extract)	TPC (mg GAE/g Extract)
Water	67.55 ± 1.09	6.53 ± 0.12
80% Ethanol	123.81 ± 1.33	10.79 ± 0.33

**Table 2:** Phytochemical composition of MBP.

No.	Name	Formula	Rt (min)	Measured *m*/*z*	Adducts	MS/MS Fragment Ions (*m*/*z*)
1	Quinic acid [27]	C_7_H_12_O_6_	0.9461	383.1199	2M−H	75.0088; 85.0294; 99.0088; 113.036; 135.0448; 155.0465; 164.072; 191.0564; 208.0613; 226.0647
2	Hinokitiol	C_10_H_12_O_2_	2.6254	165.0909	M+H	101.0087; 107.0491; 120.0808; 121.0651; 124.0243; 137.0597; 142.0347; 150.0669; 164.0718; 165.0916
3	Aucubin	C_15_H_22_O_9_	3.6226	345.1196	M−H	59.0138; 71.0139; 89.0245; 101.0244; 103.0553; 106.0422; 119.0344; 121.066; 165.0561; 345.1239
4	Puerarin	C_21_H_20_O_9_	5.1640	415.1294	M−H	268.0349; 282.0161; 295.0245; 296.0323; 297.0392; 311.0559; 338.1033; 340.0841; 355.1074; 370.1335
5	Rutin	C_27_H_30_O_16_	5.6846	611.1604	M+H	57.0342; 71.0497; 85.0288; 129.0551; 137.0226; 153.018; 165.0172; 229.0502; 257.041; 303.0494
6	Verbenalin	C_17_H_24_O_10_	5.9029	411.1284	M+Na	68.9977; 85.0291; 99.0442; 125.0232; 151.0388; 161.0607; 207.0648; 217.0505; 231.0655; 249.0754
7	Hyperoside	C_21_H_20_O_12_	5.9329	463.0884	M−H	71.0139; 101.0245; 107.0138; 151.0038; 243.0297; 255.0299; 271.0247; 300.0279; 301.0296; 463.0873
8	Aloenin	C_19_H_22_O_10_	5.9329	409.1146	M−H	59.0137; 127.0556; 163.0397; 171.0456; 188.0485; 203.0716; 204.041; 215.0351; 232.0373; 247.0614
9	Astragalin	C_21_H_20_O_11_	5.9514	449.1074	M+H	85.0289; 103.0545; 107.0493; 131.0491; 163.0751; 175.0758; 269.1171; 286.1434; 287.0547; 448.2018
10	Isorhamnetin	C_16_H_12_O_7_	6.0029	317.0653	M+H	153.0180; 228.0412; 229.0487; 245.0438; 246.0508; 257.0428; 273.0378; 274.0468; 285.0389; 317.0649
11	Morusin	C_25_H_24_O_6_	6.0447	438.1905	M+NH_4_	143.0490; 157.0647; 175.0752; 178.0854; 227.0699; 237.0909; 252.1154; 259.0962; 421.1636; 438.1900
12	Cynaroside	C_21_H_20_O_11_	6.1693	449.1075	M+H	57.0345; 59.0139; 71.0137; 99.0452; 101.0243; 125.0247; 143.0349; 331.1775; 373.1867; 475.2188
13	Eriocitrin	C_27_H_32_O_15_	6.2042	595.1685	M−H	59.0139; 65.0032; 107.014; 135.0452; 151.0039; 191.0696; 287.0565; 359.1519; 360.1542; 595.1638
14	Eriodictyol	C_15_H_12_O_6_	6.2751	289.0705	M+H	67.0547; 69.0704; 109.1013; 153.0181; 161.0599; 173.0596; 187.0754; 271.1695; 288.1979; 289.0695
15	Luteolin	C_15_H_10_O_6_	6.2884	285.0405	M−H	107.0135; 133.0295; 149.0249; 151.0038; 175.0404; 199.0394; 217.051; 257.1544; 284.127; 285.0404
16	Ononin	C_22_H_22_O_9_	6.4809	472.1599	M+ACN+H	85.0288; 207.0675; 236.069; 254.0806; 263.0576; 264.0648; 280.0585; 281.0677; 310.1066; 472.1595
17	Prunin	C_21_H_22_O_10_	6.6258	433.1149	M−H	59.0138; 65.0032; 83.0142; 107.0141; 119.0505; 151.0039; 271.0610; 313.0572; 432.2338; 433.1253
18	Diosmin	C_28_H_32_O_15_	6.6730	607.1100	M−H	59.0138; 89.0246; 227.0361; 255.0288; 271.0236; 284.0325; 285.0388; 299.0193; 373.1678; 607.1041
19	Isovitexin	C_21_H_20_O_10_	6.7012	487.1228	M+CH_3_OH+K	308.0604; 327.0789; 334.0365; 336.0555; 349.058; 351.0791; 377.0579; 395.0687; 452.0895; 467.1799
20	Tribuloside	C_30_H_26_O_13_	6.9509	595.1440	M+H	52.8999; 69.0341; 81.0338; 91.0547; 119.0492; 147.0438; 165.0546; 287.0544; 291.086; 309.0978
21	Kaempferide	C_16_H_12_O_6_	7.2449	299.0201	M−H	133.03; 171.0452; 175.041; 199.0403; 201.0199; 215.0357; 227.0345; 243.0294; 271.0246; 299.0197
22	Rhoifolin	C_27_H_30_O_14_	7.5514	577.1371	M−H	119.0505; 145.0297; 211.0404; 213.0555; 239.0352; 241.0503; 268.038; 269.0452; 414.1346; 577.1367
23	Procyanidin B2	C_30_H_26_O_12_	7.5530	579.1493	M+H	58.0658; 119.0493; 147.0439; 271.0595; 294.075; 373.1294; 401.1247; 416.1483; 418.1593; 578.1992
24	Kaempferitrin	C_27_H_30_O_14_	7.7164	633.1810	M−H	151.0049; 211.0408; 227.0352; 239.0315; 255.0299; 257.0429; 269.0457; 284.0329; 414.1344; 577.1367
25	Naringenin [28]	C_15_H_12_O_5_	7.7348	271.0610	M−H	63.024; 65.0033; 83.0139; 93.0346; 107.0138; 119.0502; 151.0038; 169.0149; 177.0197; 271.0611
26	Amygdalin	C_20_H_27_NO_11_	8.9810	475.1958	M+NH_4_	151.0755; 165.0546; 181.0858; 186.0673; 217.0858; 229.0856; 366.1457; 397.1637; 415.1758; 457.1857

**Table 3:** In vitro antioxidant activity of water and 80% ethanol MBP extracts.

Solvent	DPPH (IC_50_, μg/mL)	ABTS (IC_50_, μg/mL)
Water	115.77 ± 4.45	458.27 ± 76.50
80% Ethanol	88.14 ± 5.34	100.22 ± 6.84

**Table 4:** Topological data for 10 core targets.

No.	Target Name	Common Name	Uniprot ID	Degree	Betweenness Centrality	Closeness Centrality
1	Signal transducer and activator of transcription 3	STAT3	P40763	28	0.0657624	0.66666667
2	Mammalian target of rapamycin	MTOR	P42345	25	0.08375584	0.63291139
3	Hypoxia Inducible Factor 1 Subunit Alpha	HIF1A	Q16665	24	0.06052699	0.63291139
4	C-X-C Motif Chemokine Receptor 4	CXCR4	P61073	22	0.07601931	0.61728395
5	Kinase Insert Domain Receptor	KDR	P35968	21	0.04383475	0.6097561
6	Toll-Like Receptor 4	TLR4	O00206	21	0.13030473	0.58139535
7	Interleukin 2	IL2	P60568	21	0.06060622	0.57471264
8	Signal transducer and activator of transcription 1	STAT1	P42224	20	0.02031513	0.60240964
9	Mitogen-Activated Protein Kinase 1	MAPK1	P28482	20	0.05702655	0.57471264
10	Enhancer Of Zeste 2 Polycomb Repressive Complex 2 Subunit	EZH2	Q15910	19	0.10237625	0.55555556

**Table 5:** The top 10 CMap-predicted phytochemicals corresponding to the gene signature. raw-cs: raw connectivity score; norm-cs: normalized connectivity score; q-value: corrected *p*-value using false discovery rate.

No.	Compound	raw-cs	q-Value	norm-cs
1	Naringenin	−0.47	4.467 × 10^−16^	−1.51
2	Glycitein	−0.45	0.004	−1.45
3	Eugenol	−0.44	0.009	−1.41
4	Isoquercetin	−0.44	0.009	−1.42
5	Quercetagetin	−0.44	0.008	−1.42
6	Quercetin	−0.41	0.021	−1.31
7	Rosmarinic acid	−0.41	0.021	−1.31
8	Chaetocin	−0.41	0.020	−1.32
9	Butein	−0.40	0.029	−1.29
10	Arctigenin	−0.40	0.029	−1.29

## Data Availability

The data that support the findings of this study are available from the corresponding author upon reasonable request.

## Supplementary Materials

The following supporting information can be downloaded at https://www.mdpi.com/article/10.3390/plants14111725/s1, Figure S1: UHPLC-MS total ion chromatograms (TICs) the flower buds of *Magnolia biondii* Pamp. extract. The upper panel shows the TIC in positive ionization mode, and the lower panel shows the TIC in negative ionization mode.; Figure S2: MS/MS spectra of the 26 compounds identified in MBP; Figure S3. Mirror plots of representative MS/MS spectral matches for MBP compounds in the GNPS database (cosine > 0.7).

## Abbreviations

The following abbreviations are used in this manuscript:

MBP	The Flower Buds of *Magnolia biondii* Pamp.
CMap	Connectivity Map
TCM	Traditional Chinese Medicine
TFC	Total Flavonoid Content
TPC	Total Polyphenol Content
RE	Rutin Equivalents
GAE	Gallic Acid Equivalents
GNPS	Global Natural Products Social Molecular Networking
GO	Gene Ontology
BP	Biological Processes
CC	Cellular Components
MF	Molecular Functions
KEGG	Kyoto Encyclopedia of Genes and Genomes
C-T-D	Component–Disease–Target
PPI	Protein–Protein Interaction
STAT	Signal Transducer and Activator of Transcription
JAK	Janus Kinase
raw-cs	raw connectivity score
norm-cs:	normalized connectivity score
MD	Molecular Dynamics
RMSD	Root-Mean-Square Deviation
SASA	Solvent-Accessible Surface Area
RMSF	Root-Mean-Square Fluctuation
MM/PBSA	Molecular Mechanics/Poisson–Boltzmann Surface Area
