# Supplementary material for: Phytochemical Characterization, Antioxidant Activity, and Anti-Melanoma Mechanism of Flower Buds of Magnolia biondii Pamp
Source: Plants (Basel). 2025 Jun 5;14(11):1725. doi: 10.3390/plants14111725 (PMC12157075; doi:10.3390/plants14111725)

Figure S1. UHPLC-MS total ion chromatograms (TICs) of the flower buds of *Magnolia biondii Pamp.* extract. The upper panel shows the TIC in positive ionization mode, and the lower panel shows the TIC in negative ionization mode.

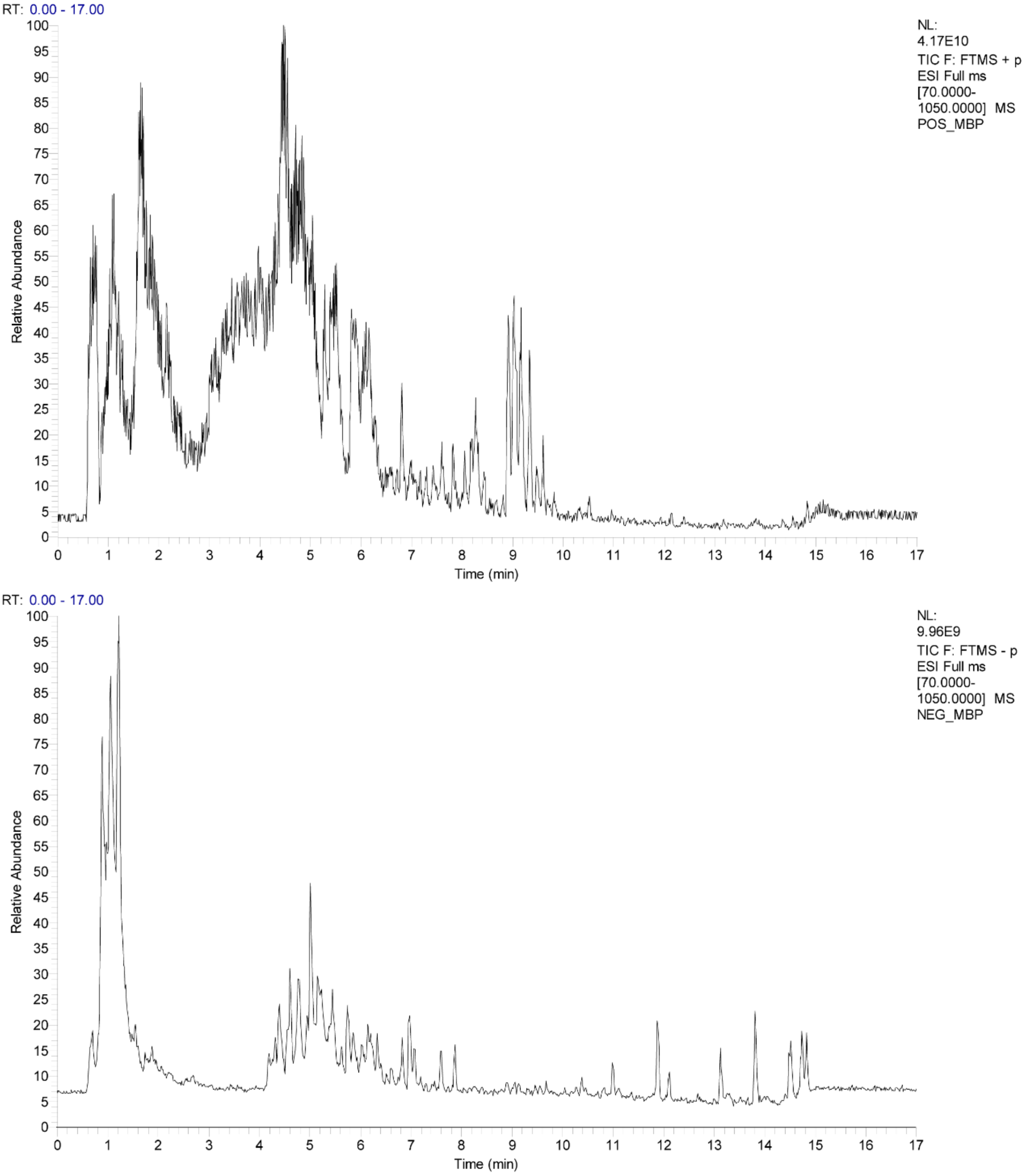

Supplement: Supplementary file 1 [file plants-14-01725-s001.zip › SI-Figure S1.pdf]
