# Supplementary material for: Phytochemical Characterization, Antioxidant Activity, and Anti-Melanoma Mechanism of Flower Buds of Magnolia biondii Pamp
Source: Plants (Basel). 2025 Jun 5;14(11):1725. doi: 10.3390/plants14111725 (PMC12157075; doi:10.3390/plants14111725)

Figure S3. Mirror plots of representative MS/MS spectral matches for MBP compounds in the GNPS database (cosine > 0.7).

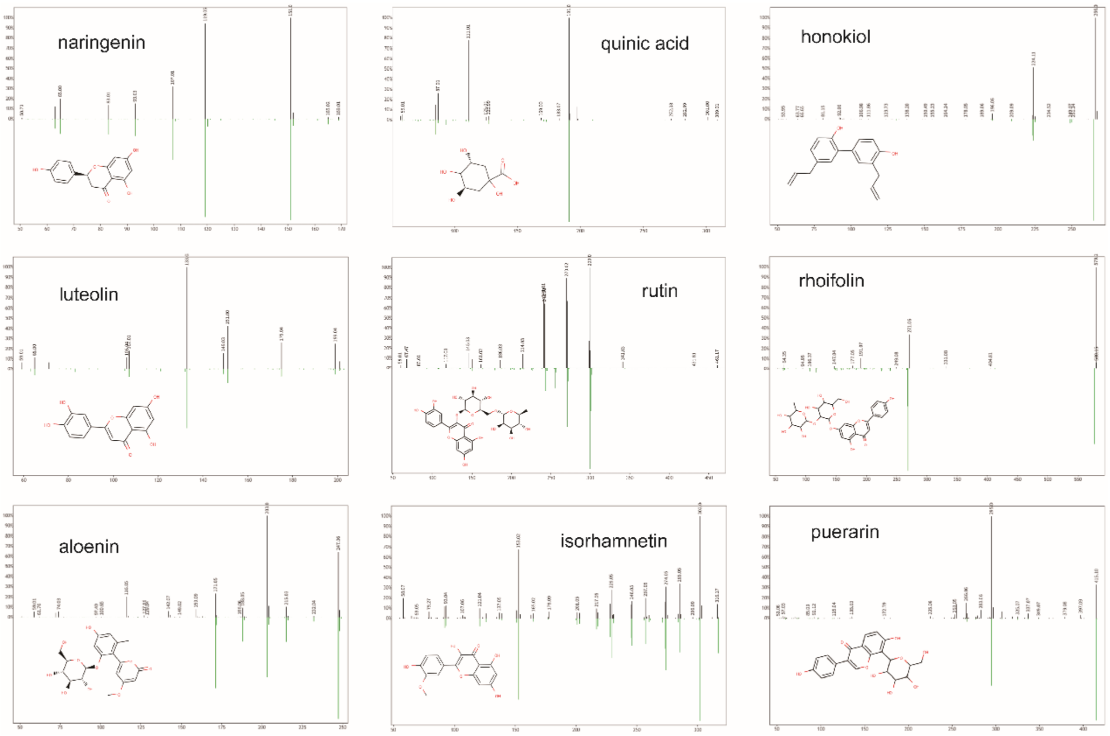

Supplement: Supplementary file 1 [file plants-14-01725-s001.zip › SI-Figure S3.pdf]
