# Supplementary figures and images for: Phytochemical Characterization, Antioxidant Activity, and Anti-Melanoma Mechanism of Flower Buds of Magnolia biondii Pamp
Source: Plants (Basel). 2025 Jun 5;14(11):1725. doi: 10.3390/plants14111725 (PMC12157075; doi:10.3390/plants14111725)

**Figure S2.** MS/MS spectra of 26 identified components of MBP.

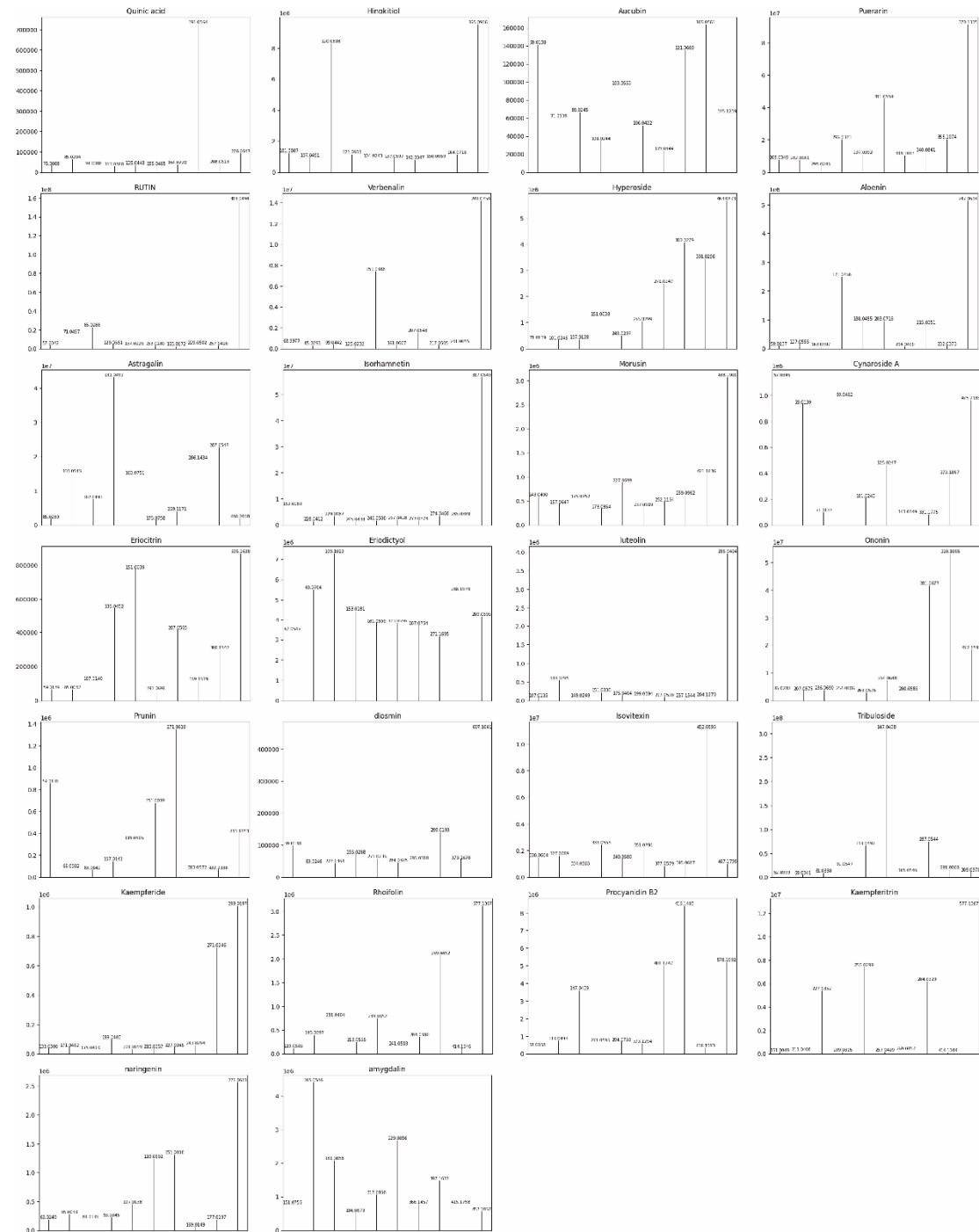

Supplement: Supplementary file 1 [file plants-14-01725-s001.zip › SI-Figure S2.pdf]
